# Hypoxic tumor-derived exosomal miR-31-5p promotes lung adenocarcinoma metastasis by negatively regulating SATB2-reversed EMT and activating MEK/ERK signaling

**DOI:** 10.1186/s13046-021-01979-7

**Published:** 2021-06-01

**Authors:** Fengqiang Yu, Mingqiang Liang, Yu Huang, Weidong Wu, Bin Zheng, Chun Chen

**Affiliations:** 1 Department of Thoracic Surgery, Fujian Medical University Union Hospital, #29 Xinquan Road, 350001 Fuzhou, Fujian China; 2 Key Laboratory of Cardio-Thoracic Surgery (Fujian Medical University), Fujian Province University, Fuzhou, China

**Keywords:** Lung adenocarcinoma, hypoxia, exosome, miR-31-5p, SATB2

## Abstract

**Background:**

Exosomes have emerged as critical mediators of intercellular communication. Hypoxia is widely recognized as a key regulator of tumor aggressiveness, and significantly affects exosome release by tumor cells. However, the effects of exosomes derived from hypoxic lung adenocarcinoma (LUAD) cells are poorly understood.

**Methods:**

Samples of miRNA isolated from hypoxic LUAD cell-derived exosomes (HExo) and normoxic LUAD cell-derived exosomes (NExo) were sequenced to identify miRNAs that might mediate tumor progression. Exosomal miRNA was co-cultured with LUAD cells to assess its biological effects on cell migration and metastasis both *in vitro* and *in vivo*. The cellular target of exosomal miRNA was confirmed by dual-luciferase assays. Western blot studies showed that exosomal miRNA regulated the related pathway. The availability of circulating exosomal miRNA derived from plasma was also evaluated.

**Results:**

We found that HExo could significantly enhance the migration and invasion of normoxic LUAD cells. MiRNA sequencing results suggested that miR-31-5p was largely internalized within HExo and could be taken up by normoxic LUAD cells. Exosomal miR-31-5p was found to directly target Special AT-Rich Sequence-Binding Protein 2 (SATB2)-revered epithelial mesenchymal transition and significantly increase activation of MEK/ERK signaling, thereby contributing to tumor progression both *in vitro* and *in vivo*. Furthermore, higher levels of circulating exosomal miR-31-5p were detected in LUAD patients, especially in patients with metastatic disease.

**Conclusions:**

Our findings demonstrate that exosomal miR-31-5p exerts a crucial role in LUAD progression, and could serve as a diagnostic biomarker for LUAD.

**Supplementary Information:**

The online version contains supplementary material available at 10.1186/s13046-021-01979-7.

## Background

Lung cancer is the highly malignant tumor with leading cancer-related mortality worldwide [1]. The most common type of lung cancer is non-small cell lung cancer (NSCLC), which can be further categorized into three subtypes: large cell lung carcinoma, lung squamous cell carcinoma and lung adenocarcinoma (LUAD). The latter is the most common type and accounts for 40 % of all lung cancers [2]. More than 50 % of LUAD patients are initially diagnosed with advanced metastatic disease, and the 5-year survival rate of those patients is only 4 % [3]. Accumulating evidence demonstrates that tumor microenvironment (TME) extremely involves in regulating tumor angiogenesis, cell proliferation, epithelial-mesenchymal transition (EMT), and even metastasis [4, 5]. Hypoxia, defined as a condition of insufficient oxygen, is a common feature of the TME and serves to accelerate tumor progression and metastasis by activating hypoxic signaling pathways [6, 7]. Increasing evidence suggests that the aberrant expression of hypoxia-related biomarkers is strongly associated with a worse clinical outcome [8]. However, it remains unclear how tumor cells communicate with their microenvironment in response to hypoxic conditions, and subsequently exhibit a more aggressive phenotype.

Exosomes comprise a class of membrane-bound extracellular vesicles with a size range of 50–150 nm. Exosomes could be released extracellularly by almost all types of cells following the fusion of multivesicular bodies or mature endosomes with the cellular membrane [9–11]. An early hypothesis supported the view that exosomes function as garage bags that eliminate nonfunctional cellular constituents. Subsequently, it was found that exosomes carry various bioactive molecules, such as proteins, lipids, and non-coding RNAs that can be transmitted to recipient cells and produce physiological or pathological effects on those cells [12]. Tumor-derived exosomes have been found to regulate cancer progression by inducing autocrine/paracrine oncogenesis, angiogenesis, TME remodeling, modulating the immune system and pre-metastatic niche formation [13, 14]. MicroRNAs (miRNAs), small class of endogenous non-coding RNAs, are major bioactive components carried by tumor-derived exosomes. MiRNAs encapsulated by exosomes regulate gene expression in recipient cells, and can thereby contribute to tumor aggressiveness and invasiveness [15].

Hypoxia has been shown to stimulate exosome release and lead to significant changes in cellular contents and functions, indicating critical effects of exosomes as regulators in distant intercellular communication [7]. Li et al [16] reported that hypoxia significantly increased the levels of exosomal miR-21 and significantly enhanced oral squamous cell carcinoma (OSCC) cell migration and invasion in HIF-1α- and HIF-2α–dependent manners. Additionally, Tadokoro et al [17] showed that hypoxic leukemia cells were responsible for the exosome-mediated transferring of miR-210 to facilitate human umbilical vein endothelial tube formation. These findings indicate that exosomes derived from hypoxic cells can alter the miRNA profiles of their target cells, and induce changes in cell phenotype.

In present study, we found that hypoxia increased the release of exosomes by LUAD cells. Hypoxic LUAD cell-derived exosomes (HExo) could enhance cell motility and metastasis. Furthermore, we examined the miRNA profiles of HExo and normoxic LUAD cell-derived exosomes (NExo). Exosomal miR-31-5p was found to be remarkably upregulated in HExo, and promotes tumor progression by targeting Special AT-Rich Sequence-Binding Protein 2 (SATB2) and activating MEK/ERK pathway. We also demonstrated that the expression of plasma-derived exosomal miR-31-5p were significantly increased in LUAD patients when compared with healthy control subjects. These data suggest that exosomal miR-31-5p is involved in tumor development and might serve as a diagnostic marker for LUAD.

## Materials and methods

### Cell culture and hypoxic exposure

The human LUAD cell lines A549, H1299, H292 and H1975 were obtained from the Cell Bank of the Chinese Academy of Sciences. All LUAD cell lines were cultured at 37℃ in a humidified incubator with a 5 % CO_2_ and 20 % O_2_ atmosphere in RPMI-1640 medium (Gibco, Waltham, MA, USA) supplemented with 10 % fetal bovine serum (FBS) and 1 % penicillin-streptomycin. For hypoxic treatment, the cells were cultured in a H35 HEPA hypoxystation (Whitley, UK) flushed with a mixture of 1 % O_2_, 94 % N_2,_ and 5 % CO_2_ for 24–48 h. Prior to exosome isolation, the cells were incubated in medium supplemented with 10 % exosome-depleted FBS obtained by overnight ultracentrifugation at 100,000 g to avoid the effects of bovine serum exosomes [18].

### Clinical samples

Samples of human blood plasma from 82 LUAD patients and 39 healthy control subjects, as well as 50surgically resected LUAD tissues were collected at Fujian Medical University Union Hospital (Fuzhou, Fujian, P.R. China). All the subjects provided their written informed consent, and the study protocol was approved by the Ethics Committee of Fujian Medical University Union Hospital.

### Exosome isolation, purification. and characterization

Exosomes were isolated from LUAD cell-derived supernatants as previously described [19]. The isolation method included a penultimate ultracentrifugation step (100,000 g for 70 min at 4 °C) needed to obtain the exosomes pellets, which were then washed with phosphate-buffered saline (PBS) and subjected to a second ultracentrifugation at 100,000 g for 70 min at 4 °C. When isolating exosomes from the plasma of LUAD patients, 1 mL of 0.8 μm-filtered blood plasma was purified by using Exosupur columns (Echo biotech, China) and a combination of size-exclusion chromatography (SEC) and ultrafiltration [20]. The final pellets were resuspended in PBS for use in subsequent experiments. The quantities of exosomes isolated for use both *in vivo* and *in vitro* experiments were determined using a BCA Protein Assay Kit (Beyotime, Shanghai, China). For transmission electron microscope (TEM) studies, purified exosomes pellets were fixed with 4 % paraformaldehyde (PFA) and placed onto copper mesh Formvar-coated grids. The grids were then stained with 2 % phosphotungstic acid for 5 min. The samples were observed using a JEOL TEM (JEM1230, JEOL, Tokyo, Japan). The size distribution and concentration of exosomes were detected by Nanoparticle Tracking Analysis (NTA). The data were analyzed with a ZetaView PMX 110 Nanoparticle Analyzer (Particle Metrix, Meerbusch, Germany) and the corresponding software ZetaView 8.04.02.

### Scratch assays

Cells were seeded on each side of an Ibidi culture insert (Ibidi, Munich, Germany) with a separation between each side of the well, and allowed to create a confluent monolayer. The cells were then treated with exosomes (10 µg/mL) for 2 h following removal of the insert, and were cultured with 1 % exosome-depleted FBS [21]. The first image (0 h) was obtained and then cultured in a 37℃ incubator for 24 h prior to acquisition of the second image. Percent of wound closure was calculated as: migrated cell surface area/total surface area × 100.

### Cell invasion and migration assays

Cell invasion assays were performed in triplicate using a 24-well Transwell plate (Corning, NY, USA) coated with Matrigel (Corning). The cell migration assays were constructed using a similar protocol, but without the Matrigel coating. Cells were treated with 10 µg of exosomes for 2 h and then seeded in the upper chamber of a Transwell plate containing serum-free RPMI-1640 medium. The bottom chamber was filled with 10 % FBS medium. After 24 h of incubation, migrated or invaded cells to the bottom of the insert membrane were fixed with 4 % paraformaldehyde and stained with 1 % crystal violet.

### RNA extraction and real-time quantitative polymerase chain reaction (RT-qPCR) analysis

The total RNA of cells and total miRNA of exosomes were extracted using TRIzol reagent (Invitrogen, Carlsbad, CA, USA) and a miRNeasy Mini Kit (Qiagen, Germany), respectively. Prior to the isolation of exosomal RNA, 25 fmol of *C. elegans* cel-miR-39 standard RNA (RiboBio, Guangzhou, China) was added to each sample as a spike-in control [22]. cDNA synthesis was performed using a PrimeScript™ RT reagent kit (Takara, Shiga, Japan) according to the manufacturer’s instructions. The miRNAs were reverse transcribed using a Mir-X miRNA First-Strand Synthesis Kit (Takara, Shiga, Japan). RT-qPCR was performed using SYBR Green GoTaq Master Mix (Promega, Madison, WI, USA) and the products were analyzed on an ABI 7500 thermocycler (Applied Biosystems, Foster City, CA, USA). The relative levels of mRNA and miRNA in cells were normalized to those of β-actin and U6, respectively, and the levels of exosomal miR-31-5p was normalized to those of cel-miR-39.

### GW4869 and RNase A treatment

To block exosomes release, LUAD cells exposed to hypoxic condition were pretreated with GW4869 (an inhibitor of exosome formation and release, Sigma-Aldrich, St. Louis, MO USA) at a concentration of 10 µM for 24 h. For RNase A treatment, exosomes were incubated with RNase A (Takara, Shiga, Japan, final concentration of 10 µg/mL) alone or combined with 0.1 % Triton X-100 for 20 min.

### Library preparation and sequencing

A 100 ng sample of total RNA was ligated with sequencing adapters using a TruseqTM Small RNA sample prep Kit (Illumina, San Diego, CA, USA), and then reversed transcribed to cDNA and PCR amplified. sRNA sequencing libraries were prepared using a TruSeq Small RNA Sample Preparation Guide (Illumina, San Diego, CA, USA) at Shanghai Majorbio Bio-pharm Technology Co. Ltd, and sequenced on an Illumina HiSeq platform. For quality control, low-quality bases (Sanger base quality of < 20) of the 3’ end were trimmed using in-house perl scripts. All identical sequences of sizes ranging from 18 to 32 nt were counted and eliminated from the initial data set. Through a BLAST search of the miRbase and the Rfam database, perfectly matched sequences were used to count, and then remove non-miRNA sequences (rRNA, tRNA, snoRNA, etc).

### Western blot analysis

Total proteins were isolated with RIPA lysis buffer (Beyotime) and quantified with a BCA kit. An equal amount of protein lysate from each sample was separated on SDS-polyacrylamide gels, and the protein bands were transferred onto polyvinylidene difluoride membranes that were subsequently blocked with 5 % skimmed dry milk. Next, the membranes were incubated with the following antibodies: anti-HIF-1α (Hypoxia Inducible Factor 1 Subunit Alpha, Abcam, Cambridge, MA, USA), anti-CD63 (Abcam), anti-TSG101 (Tumor Susceptibility 101, Abcam), anti-GAPDH (glyceraldehyde 3-phosphate dehydrogenase)anti-Flotillin (Abcam), anti-SATB2 (Abcam); anti-phosphorylated (p)-MEK (Abcam), anti-MEK (Abcam), anti-phosphorylated (p)-ERK (Affinity Biosciences, Cincinnati, OH, USA), anti-ERK (Affinity); anti-E-cadherin (Cell Signaling Technology, Danvers, MA, USA), anti-N-cadherin (Cell Signaling Technology), anti-Vimentin (Cell Signaling Technology) at 4℃ overnight and then incubated with a secondary antibody for 1 h at room temperature. β-actin (Abcam) and Calnexin (Boster, Wuhan, China) were served as a loading control and a negative control for exosomal markers, respectively. The chemiluminescent signals were detected and the immunostaining intensity of each band was quantified using a Bio-Rad ChemiDoc XRS system (Bio-Rad, Hercules, CA, USA).

### Animal studies

Athymic nude mice (male, 5 weeks of age) were purchased from the SLUAD Laboratory Animal, Shanghai, China, and housed in a specific pathogen-free environment. Twenty mice were randomly assigned to four groups (5 mice per group). A549 cells were intravenously injected into the tail vein of each mouse (2 × 10^6^ cells/200 µL/per mouse) to establish lung metastatic model. Next, equal amounts (10 µg) of purified exosomes were also intravenously injected into the tail vein of each mouse twice per week for 4 weeks. All the mice were sacrificed after 8 weeks, and their lungs were collected and subjected to hematoxylin and eosin (HE) staining. The numbers of metastatic nodules in the lung tissues were also counted. The protocols for all animal studies were approved by the Ethics Committee of the Animal Center of Fujian Medical University.

### Luciferase activity assay

The 3’UTR segments of SATB2 genes were amplified by the PCR and inserted into SATB2 vectors. Co-transfection with the vectors and miR-31-5p mimics was performed using Lipofectamine 3000 (Invitrogen). Luciferase activity was measured after 36 h of incubation using a Dual-Luciferase Reporter Assay Kit (TransGen Biotech, Beijing, China) as described in the manufacturer’s instructions.

### Cell transfection and internalization of exosomes

SATB2 siRNA, miR-31-5p mimics and inhibitors were obtained from RiboBio Company (Guangzhou, China). Cells were seeded into 6-well plates for 24 h before transfection. When the cells reached 60 % confluence, they were transfected with SATB2 siRNA, miRNA mimics or inhibitors using Lipofectamine 3000. To determine whether the exosomes could be internalized by LUAD cells, the exosomes were labeled with PKH67 (Sigma) as the manufacturer described. Briefly, purified exosomes were suspended in a mixture consisting of 500 µL of Diluent C and 4 µL of PKH67, and then incubated in the dark for 4 min at room temperature. Next, 2 mL of 0.5 % bovine serum albumin (BSA) was added to stop staining, and the mixture was subsequently centrifuged at 100,000 g for 1 h. The labeled exosomes were suspended with PBS and then used immediately or stored at 20℃. LUAD cells were treated with labeled exosomes for 8 h at 37℃, and the nuclei were stained by Dapi (Sigma) for 30 min. After staining, the samples were washed twice with PBS and observed under a fluorescence microscope.

### Immunohistochemistry

Samples of metastatic lung tissue from each mouse were fixed in 10 % formalin and paraffin embedded. Section (4 μm thickness) were placed onto glass slides. The slides were incubated at 37℃ with mouse anti-E-cadherin and mouse anti-Vimentin for 2 h; Slides then were incubated with HRP-conjugated anti-mouse antibody for 30 min and stained with DAB.

### Statistical analysis

All the statistical analyses were performed using IBM SPSS Statistics for Windows, Version 22.0 software (IBM Corp, Armonk, NY, USA). Graphpad Prism 8.0 software (Graphpad Prism Software, Inc, San Diego, CA, USA) was used to create graphs. Results are presented as a mean value ± standard deviation. One-way analysis of variance or the unpaired Student’s t-test was used for statistical analysis. Relationships between exosomal miR-31-5p and other factors were analyzed using the χ^2^ test. Receiver operating characteristic (ROC) curves were generated to assess the diagnostic value of exosomal miR-31-5p. The association between SATB2 and miR-31-5p was explored by Pearson’s correlation. A *P*-value<0.05 was considered to be statistically significant.

## Results

### Identification of exosomes derived from LUAD cell conditioned medium

**Fig. 1:**
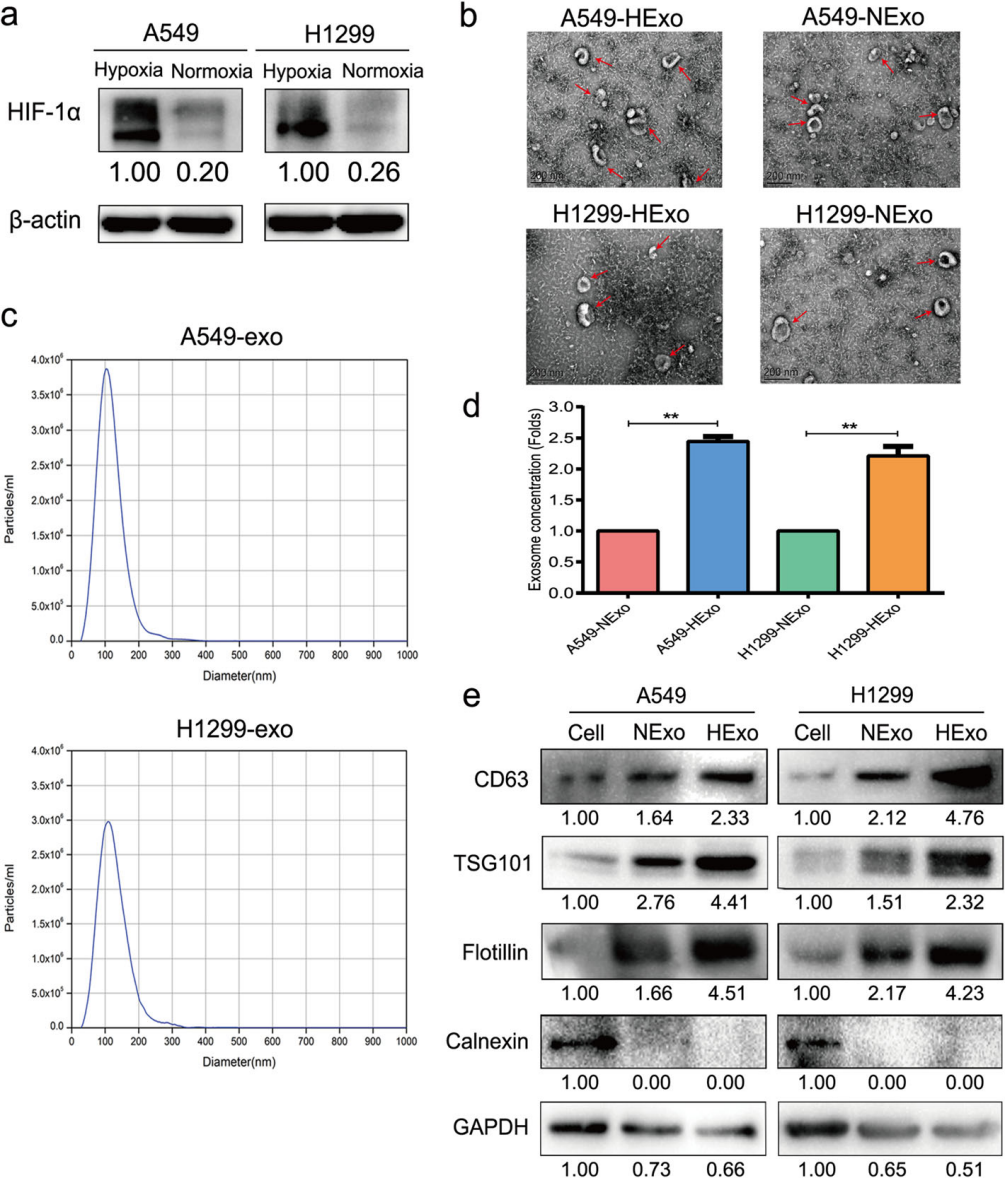
Characterization of exosomes derived from LUAD cells.** a** Expression of HIF-1α in A549 and H1299 cells under hypoxic and normoxic conditions. **b** Representative TEM picture of exosomes derived from LUAD cells. **c** NTA were detected the concentration and size distribution of HExo. **d** Comparison of exosomes concentration between HExo and NExo. **e** Western blot analysis for presence of exosomes markers, CD63, TSG101, Flotillin and absence of negative control, Calnexin. Data are presented as the mean ± SD of three independent experiments (**, *P*<0.01)

LUAD cells were cultured under hypoxic (1 % O_2_) or normoxic (20 % O_2_) conditions for 48 h. We found that the levels of HIF-1α expression were significantly increased in hypoxic conditions (Fig. 1a), indicating that we had successfully established a hypoxic model for use in subsequent experiments. Next, exosomes derived from LUAD conditioned medium were isolated by ultracentrifugation. The morphology of the purified exosomes was observed by TEM, which showed typical rounded lipid bilayer particles with a size distribution of 50–150 nm (Fig. 1b). NTA data further confirmed that most of the particles were ~ 100 nm in diameter (Fig. 1c), and the concentration of exosomes released under hypoxic conditions was much higher than that under normoxic conditions (Fig. 1d). Moreover, we sought to verify the presence of exosomal markers on the purified exosomes by western blot, which revealed that CD63, TSG101 and Flotillin were highly expressed in the exosomes, and Calnexin was used as a negative control (Fig. 1e). These results indicated that we were capable of isolating purified exosomes, and hypoxia boosted secretion of exosomes.

### HExo enhanced the migration and invasion capabilities of normoxic cells

**Fig. 2:**
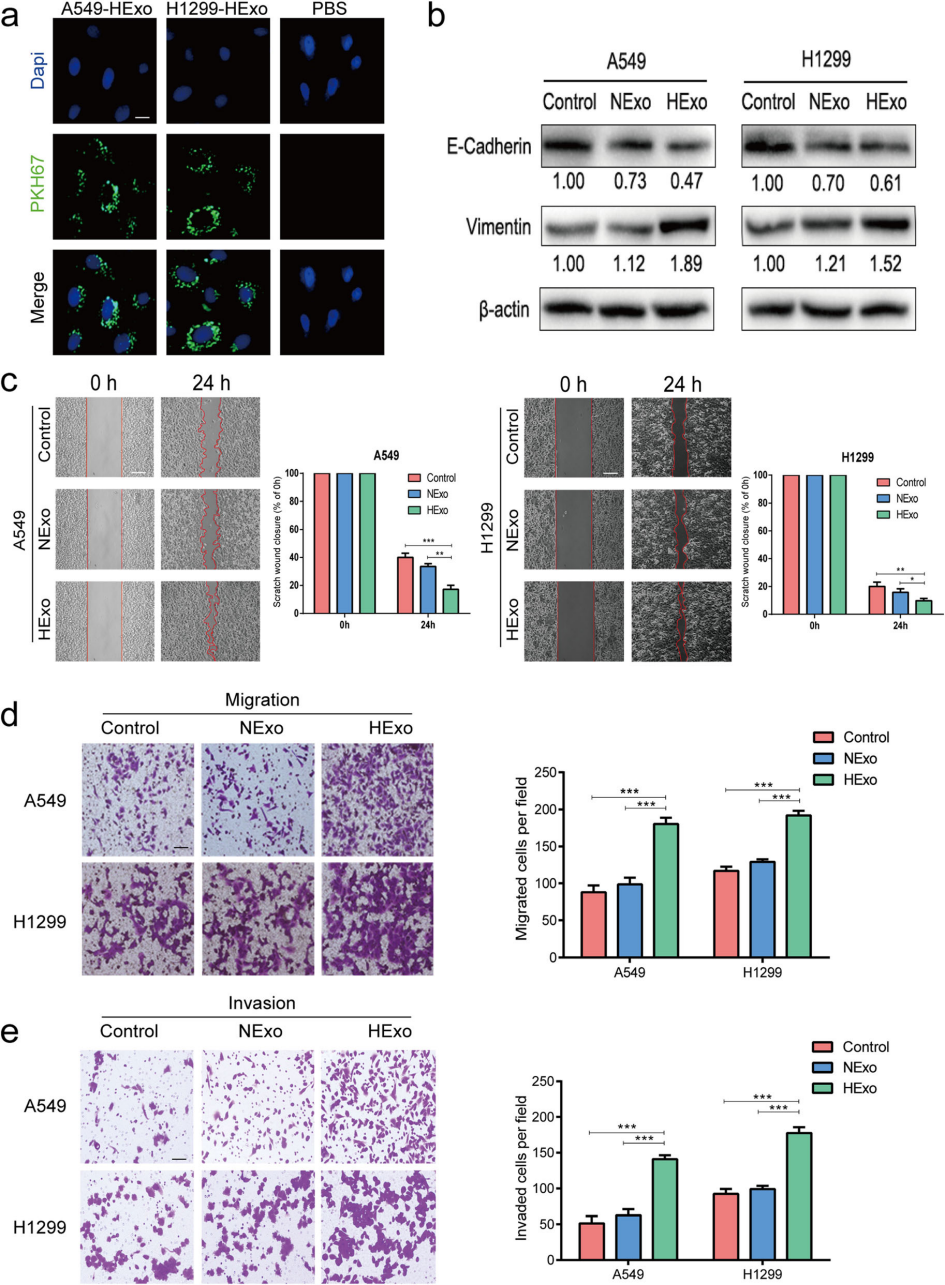
Effects of HExo on LUAD. **a** Internalization of PKH67-labeled exosomes by LUAD cells. Scale bar, 20 μm. **b** Western blot analysis for E-cadherin and Vimentin in the control (PBS), NExo and HExo groups. **(c-d)** Scratch assays and migration assays were assessed in the control (PBS), NExo and HExo groups, respectively. Scale bar, 20 μm. **(e)** Invasion assays were analyzed in the control (PBS), NExo and HExo groups. Scale bar, 20 μm. Data are represented as the mean ± SD from three independent experiments (*, *P*<0.05; **, *P*<0.01; ***, *P*<0.001)

To explore the biological effects of HExo on LUAD cells, A549 and H1299 cells were treated with HExo and then observed for any phenotypic changes. The HExo were labeled with fluorescent PKH67, which would allow us to confirm their internalization by LUAD cells as detected by fluorescent microscopy (Fig. 2a).We detected the EMT-related markers changes and found that HExo significantly decreased E-cadherin expression while increasing Vimentin expression (Fig. 2b). When compared with the NExo and PBS control groups, scratch assays revealed that tumor cells treated with HExo dramatically decreased the amount of open space (Fig. 2c). Similarity, HExo treatment promoted LUAD cell migration in transwell assays (Fig. 2d). In addition to the migration assay results, LUAD cells co-cultured with HExo showed a remarkably increased invasive ability (Fig. 2e). These data indicated that HExo could facilitate the migration and invasion of normoxic cells *in vitro*.

### HExo-mediated transfer of miR-31-5p to normoxic cells

**Fig. 3:**
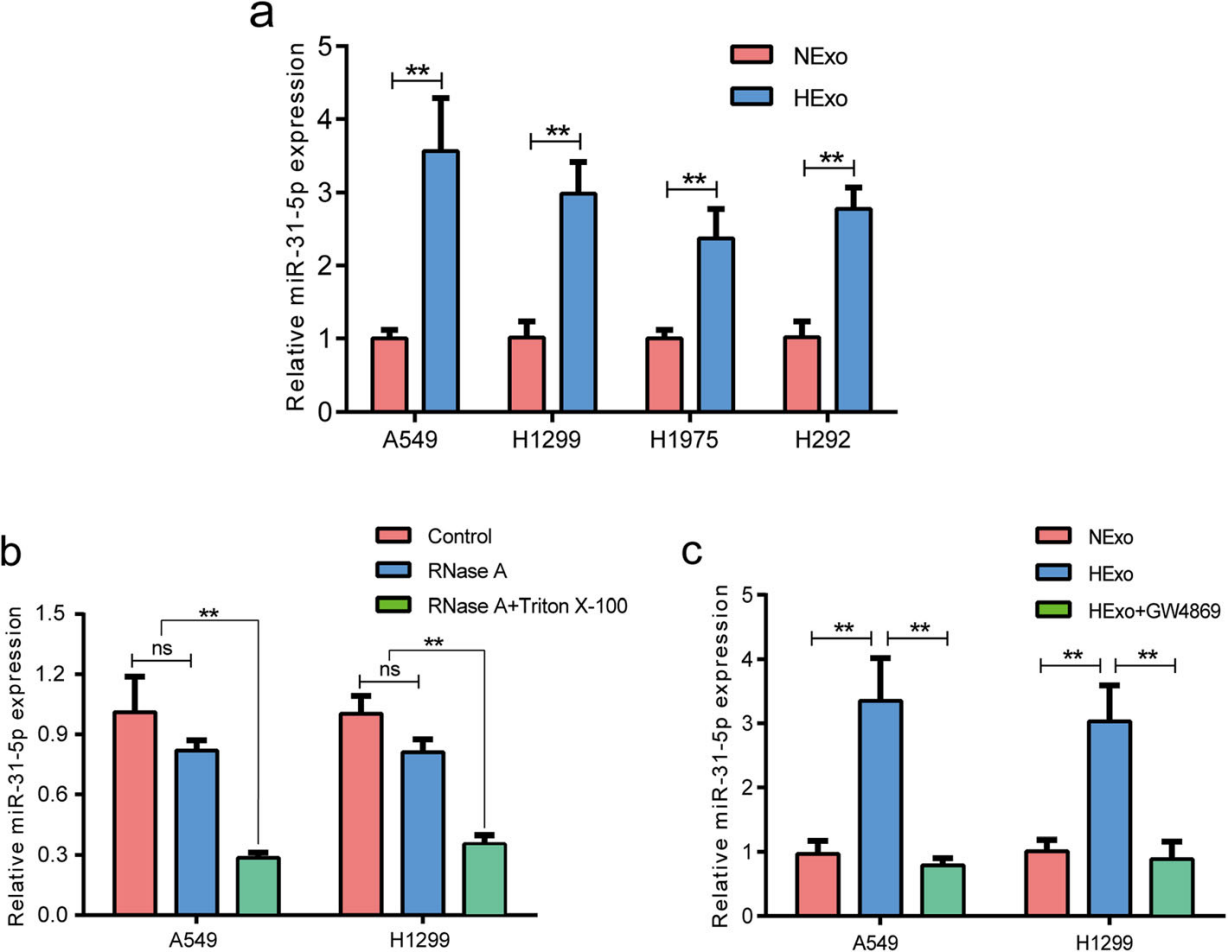
miR-31-5p was encapsuled in HExo. **a** Expression of exosomal miR-31-5p in HExo and NExo of four LUAD cell lines. **b** Expression of exosomal miR-31-5p in nontreated, RNase A-treated and RNase A + Triton X-100-treated groups. **c** Expression of exosomal miR-31-5p in NExo, HExo and HExo + GW4869 groups. Data are represented as the mean ± SD from three independent experiments (**, *P*<0.01)

A previous study confirmed that exosomes containing miRNAs could be transferred to recipient cells and influence cellular function [23]. To investigate whether this transfer could occur in LUAD cells, total RNA of HExo and NExo was extracted for miRNA-seq analysis by using the Illumina HiSeq platform. After a series of quality control, the expression level of each miRNA was calculated according to the transcripts per million (TPM) reads method. The data showed that 131 overlapping known miRNAs were simultaneously identified in both groups (Additional file 1: Fig. S1a). Additionally, miRNAs that showed significant differential expression (DE) were identified by DEseq2 based on |log2FC| >1 and FDR < 0.05 criteria. We discovered that the expression of 15 known miRNAs were extremely upregulated in the HExo groups (Additional file 1: Fig. S1b). Next, we designed primers for miRNAs and validated the accuracy of miRNA-seq using RT-qPCR. Consistent with the miRNA-seq results, our data verified that miR-21-5p, miR-31-5p, miR-210-3p, miR-21-3p and miR-181a-5p were significantly upregulated both in A549 and H1299 (Additional file 1: Fig. S1c). Because miR-31-5p was expressed at highest level, and it also stably upregulated in other two LUAD cells lines (H292 and H1975) (Fig. 3a). These data exhibited good repeatability as compared with other miRNAs. Moreover, miR-31-5p was considered as a oncogenic factor in other types of cancer. So we chose miR-31-5p for further studies.

To confirm whether miR-31-5p was encapsulated within exosomes, we added RNase A, which can degrade free RNA, and Triton X-100, which increases cell membrane permeability. As expected, the levels of miR-31-5p expression in the RNase A treatment groups were consistent with those in the control group. However, the miR-31-5p levels significantly decreased in both the RNase A and Triton X-100 groups (Fig. 3b). These results revealed that miR-31-5p was transmitted to other cells by LUAD cell-derived exosomes and protected from degradation by being encapsulated in double lipid bilayers. Furthermore, we also found that hypoxic conditions could lead to more elevated levels of miR-31-5p expression in HExo than in NExo, but those levels were reversed by GW4869 treatment (Fig. 3c), which further indicated that hypoxic conditions promoted the secretion of exosomes and the transport of miR-31-5p to recipient cells.

### Exosomal miR-31-5p promoted LUAD cell migration and invasion by directly targeting SATB2

**Fig. 4:**
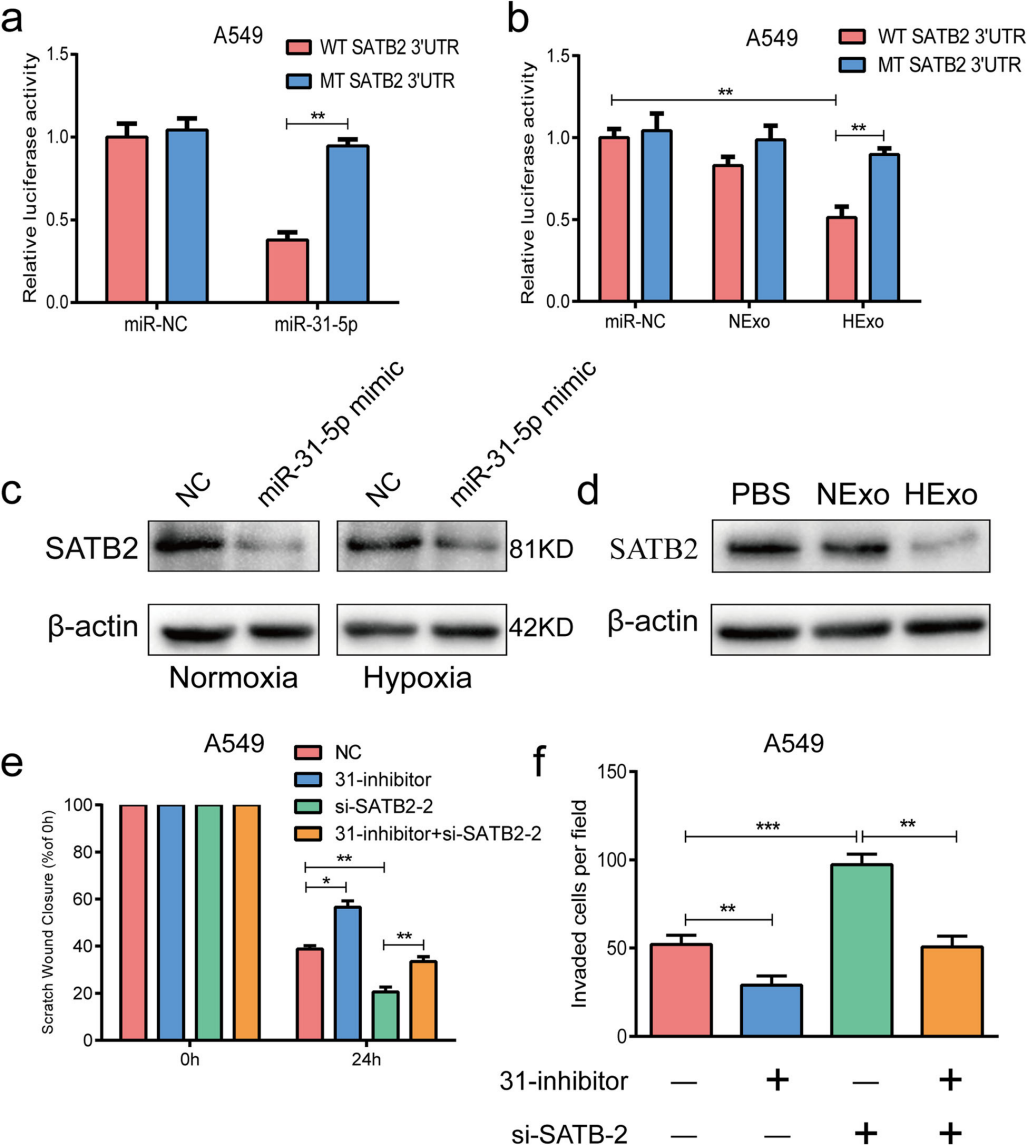
Exosomal miR-31-5p directly targeted SATB2. **a-b** Luciferase activities of WT or mut 3’UTR in A549 cells after transfection of miR-31-5p mimic or HExo. **c** Expression of SATB2 after transfection miR-31-5p mimic under hypoxic or normoxic conditions. **d** Expression of SATB2 after treatment of HExo. Scratch assays (**e**) and Transwell invasion assays (**f**) results after transfection of si-SATB2 and/or miR-31-5p inhibitor in A549 cell line. Data are represented as the mean ± SD from three independent experiments (*, *P*<0.05; **, *P*<0.01; ***, *P*<0.001)

To identify the possible cellular target of miR-31-5p, we conducted an overlap analysis by using three online databases: Targetscan, miRTarbase, and miRDB (Additional file 2: Fig. S2a). The analysis suggested SATB2 as the target of miR-31-5p (Additional file 2: Fig. S2b). The efficiencies of the miR-31-5p mimic and inhibitor were detected by RT-qPCR (Additional file 2: Fig. S2c). Next, luciferase assays were performed to verify the predictive accuracy of the database analysis. Plasmids containing the wild type (WT) or mutant type (Mut) 3’UTR sequence of SATB2 with the predicted miR-31-5p binding site were cloned into a luciferase reporter. We found that the luciferase activity of A549 cells transfected with miR-31-5p mimics was remarkably reduced when compared to luciferase activity in the NC groups (Fig. 4a). Furthermore, similar results were observed in the HExo treatment groups (Fig. 4b). Additionally, cells transfected with the miR-31-5p mimics under normoxic or hypoxic conditions showed significantly reduced levels of SATB2 expression (Fig. 4c), and treatment with HExo also reduced SATB2 expression (Fig. 4d). Thus, we confirmed that SATB2 was the direct target of miR-31-5p.

We next treated LUAD cells with si-SATB2 and/or miR-31-5p inhibitor to assess whether exosomal miR-31-5p might promote a more aggressive phenotype by targeting SATB2. The knockdown efficiency of si-SATB2 was detected by western blotting (Additional file 2: Fig. S2d), which indicated that the si-SATB2-2 groups had the most significantly decreased levels of SATB2 expression. Therefore, those groups were used for further experiments. When compared with control groups, the miR-31-5p inhibitor groups displayed strongly decreased levels of activity in healing assays and Transwell invasion assays, while the si-SATB2-2 treatment groups exhibited the opposite trends in those assays. However, the migration and invasion abilities of cells in the si-SATB2-2 groups could be attenuated by treatment with the miR-31-5p inhibitor in A549 and H1299 cell lines (Fig. 4e-f and Additional file 2: Fig. S2e-f).

### Exosomal miR-31-5p facilitated metastasis *in vivo*

**Fig. 5:**
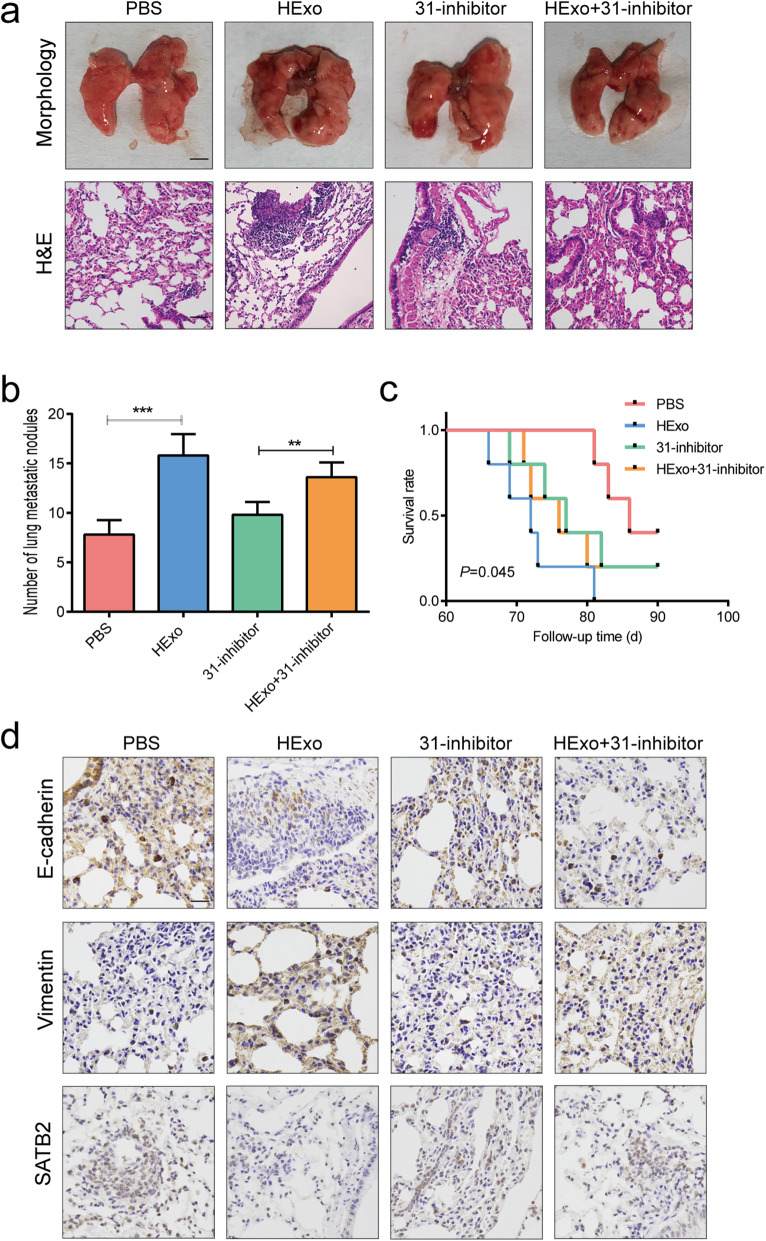
Exosomal miR-31-5p promoted metastasis of LUAD cells ***in vivo***. **a** Representative images of lung metastasis in nude mice after tail intravenous injection of A549 cells treated with HExo or miR-31-5p inhibitor (31-inhibitor) or treated with HExo and miR-31-5p inhibitor (HExo + 31-inhibitor). Scale bar, Morphology 500 mm; HE 50 μm. **b** Numbers of metastatic lung nodules in each group. **c** Overall survival times of each group after tail intravenous injection of A549 cells. **d** Representative pictures of E-cadherin, Vimentin and SATB2 expression in lung metastatic nodule by immunohistochemistry staining. Scale bar, 50 μm

We hypothesized that exosomal miR-31-5p derived from HExo could increase the metastatic ability of LUAD cells in a xenograft model. During an 8-week observation period after intravenous tail injection, we obtained samples of lung tissue from sacrificed mice and stained them with HE (Fig. 5a). After counting the numbers of metastatic nodules in the stained tissues, we found that mice in the HExo groups had more lung metastatic nodules than mice in the PBS group, and transfection with the miR-31-5p inhibitor could abrogate that effect. In contrast, treatment with HExo and the miR-31-5p inhibitor increased the numbers of metastatic nodules when compared with those numbers in the miR-31-5p inhibitor groups (Fig. 5b). We also evaluated the overall survival times of mice that received the treatments of different exosomes. Surprisingly, mice in the HExo groups had shorter overall survival times than mice in the other three groups (Fig. 5c). Furthermore, we examined the expression of EMT-related markers and SATB2 in lung tissues using immunochemistry methods. As expected, increased levels of Vimentin expression and decreased levels of E-cadherin and SATB2 expression were detected in the HExo groups, and those changes in expression were attenuated by the miR-31-5p inhibitor (Fig. 5d).

### HExo-induced progression via MEK/ERK signaling could be reversed by the miR-31-5p inhibitor

**Fig. 6:**
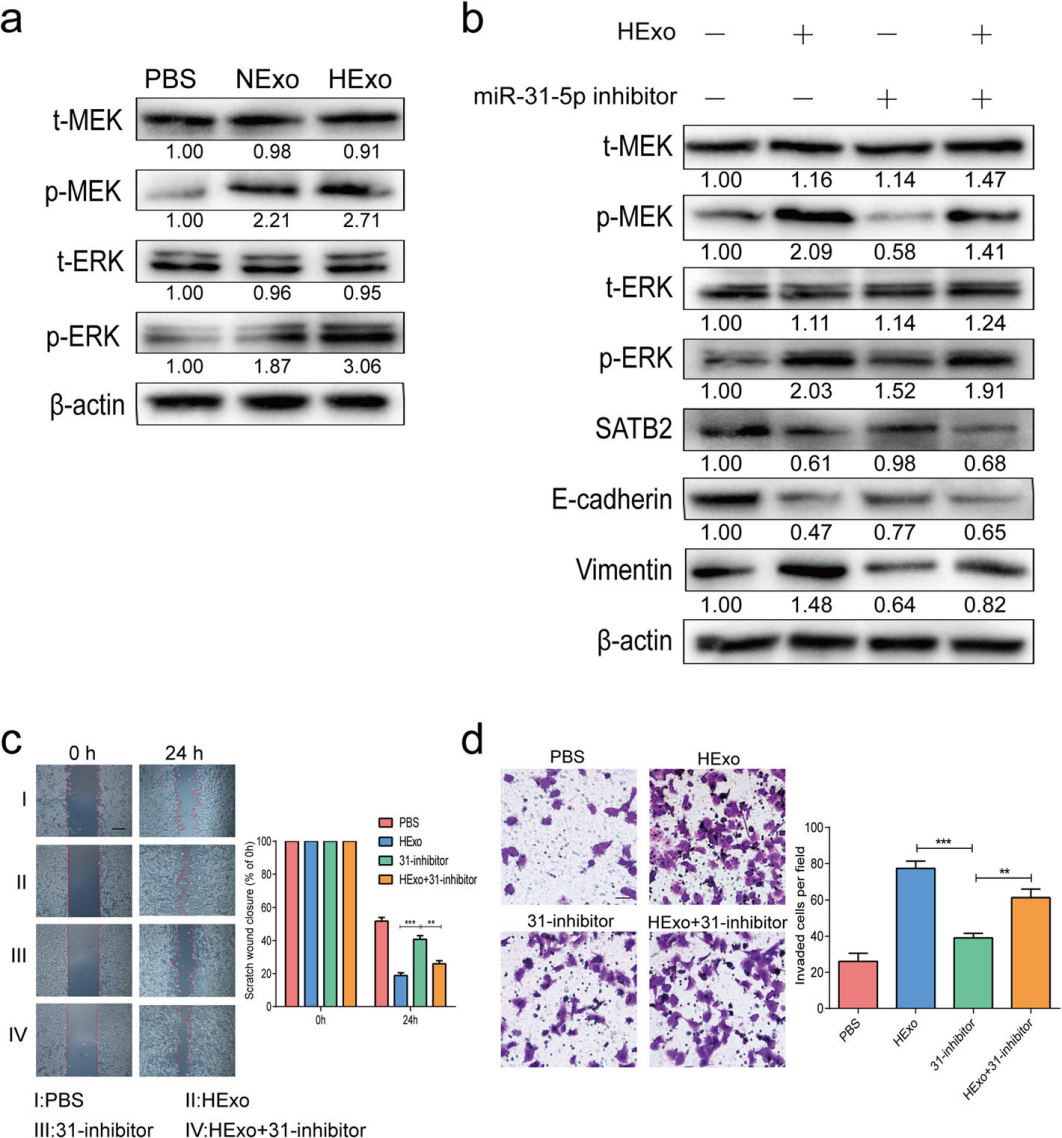
Exosomal miR-31-5p activated MEK/ERK signaling pathway. **a** Phosphorylation of MEK and ERK were determined after treatment of HExo. **b** EMT markers, E-cadherin and vimentin, phosphorylation of MEK and ERK were determined after treatment of HExo and/or miR-31-5p inhibitor. Scratch assays (**c**) and Transwell invasion assays (**d**) results after treatment of HExo and/or miR-31-5p inhibitor. Scale bar, 20 μm. Data are represented as the mean ± SD from three independent experiments (**, *P*<0.01; ***, *P*<0.001)

The MEK/ERK signaling pathway has been shown to be involved in development of aggressive lung cancer phenotypes and the EMT process [24, 25]. Therefore, to investigate the effects of exosomal miR-31-5p derived from HExo might affect MEK/ERK signaling pathway, we evaluated the phosphorylation levels of MEK and ERK. As shown in Fig. 6a, obviously enhanced phosphorylation levels of MEK and ERK were detected in the HExo groups when compared with the NExo and PBS groups. However, the effects of elevated MEK/ERK signaling activity and EMT were abolished by transfection with the miR-31-5p inhibitor, while the simultaneous addition of HExo and the miR-31-5p inhibitor could reverse those effects (Fig. 6b). We also observed a similar role played by exosomal miR-31-5p in functional experiments. Transfection with miR-31-5p inhibitor suppressed the migration and invasion abilities of LUAD cells, while treatment with HExo and miR-31-5p inhibitor reverse those capabilities (Fig. 6c-d). These results verified that exosomal miR-31-5p derived from HExo could enhance both EMT process and MEK/ERK signaling activity.

### Circulating exosomal miR-31-5p might be a diagnostic biomarker for LUAD

**Fig. 7:**
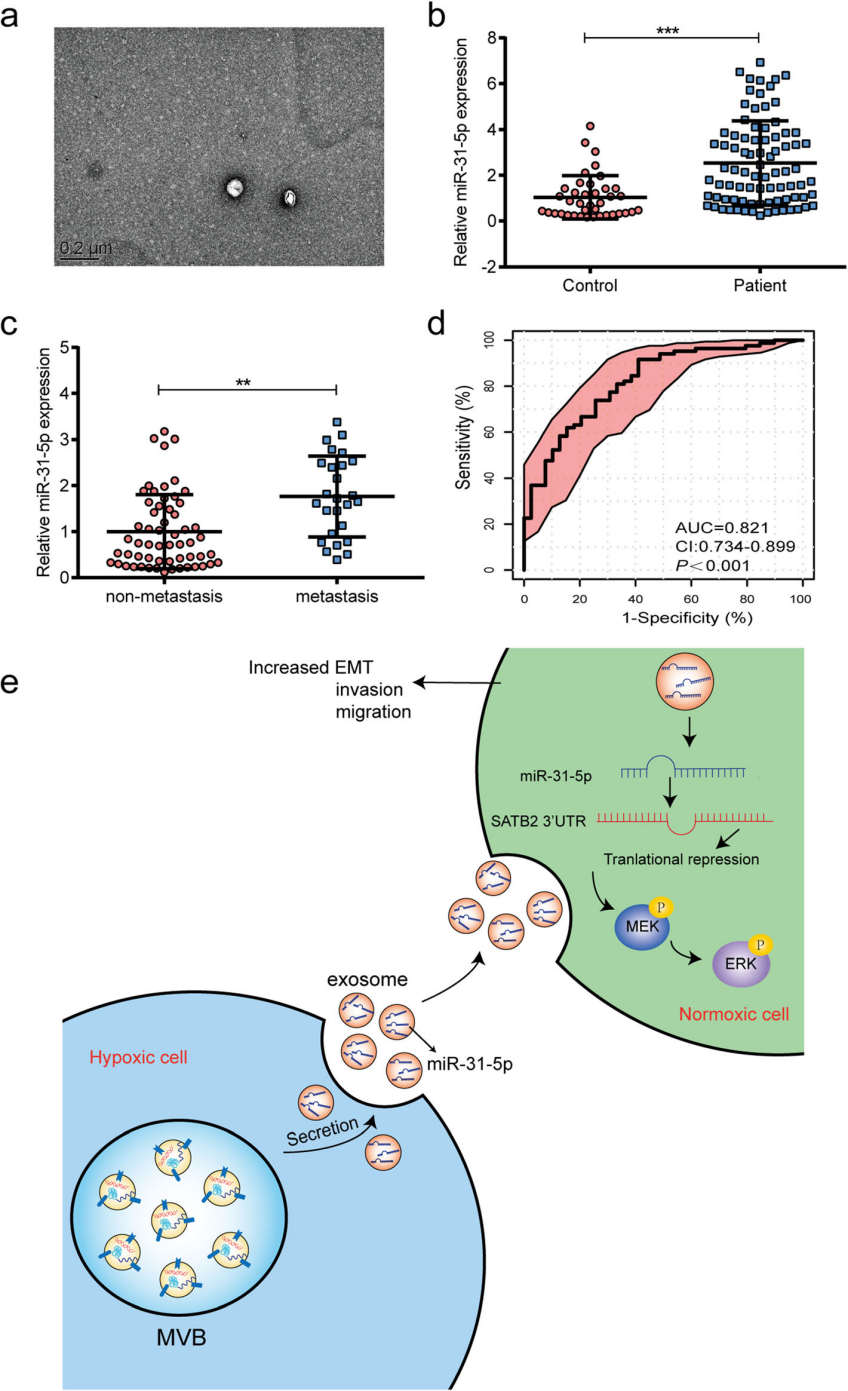
Exosomal miR-31-5p might act as a diagnostic biomarker for LUAD.** a** A representative TEM image of plasma-derived exosomes from lung adenocarcinoma patients. **b** Expression of exosomal miR-31-5p in plasma-derived from lung adenocarcinoma patients and healthy controls. **c** Expression of exosomal miR-31-5p from plasma in metastatic patients and non-metastatic ones. **d** ROC curve for plasma-derived exosomal miR-31-5p. **e** Schematic diagram of hypoxic exosomes containing miR-31-5p transferred to normoxic cells contributing to LUAD invasion and migration by negatively regulating SATB2-reversed EMT and activating MEK/ERK signaling

Studies in other types of cancer have proven that exosomes containing miRNAs derived from primary tumors can be transferred into the body’s circulation [26, 27]. To explore whether circulating exosomal miR-31-5p might be of diagnostic value for LUAD, we collected samples of blood plasma from 82 LUAD patients and 39 healthy individuals, and then isolated the exosomes from the plasma by using a combination of size-exclusion chromatography and ultrafiltration. TEM pictures showed that the plasma samples contained intact membranous particles (Fig. 7a). NTA results showed the concentration and size distribution of the particles (Additional file 3: Fig. S3a) and western blot studies revealed the presence of exosome-related markers (Additional file 3: Fig. S3b). Next, we extracted the miRNA from the plasma-derived exosomes, and found that the levels of exosomal miR-31-5p in exosomes from LUAD patients were significantly higher than those in exosomes from healthy control subjects (Fig. 7b). Furthermore, we found that the levels of exosomal miR-31-5p in all samples of LUAD plasma-derived exosomes from patients with metastatic disease were much higher than the levels in patients with non-metastatic disease (Fig. 7c), which was consistent with our verification in tissue samples (Additional file 3: Fig. S3c). We also categorized LUAD samples into high miR-31-5p expression group and low miR-31-5p expression group to analyze the association between exosomal miR-31-5p and clinicopathological characteristics. The results indicated that a high level of exosomal miR-31-5p was strongly correlated with N (*P* = 0.013) and TNM stage (*P* = 0.001, Additional Table S1). A ROC analysis of exosomal miR-31-5p showed that exosomal miR-31-5p had a high power for discriminating between LUAD patients and healthy individuals with an AUC of 0.821 (Fig. 7d). Moreover, We evaluated the discriminated power between non-metastatic patients and metastatic ones in miR-31-5p group, SATB2 group and miR-31-5p + SATB2 group through ROC analysis. We observed that miR-31-5p could be the better biomarker to predict cancer metastasis in clinics with AUC of 0.844, AUC of SATB2 + miR-31-5p group and SATB2 group is 0.835 and 0.767, respectively (Additional file 3: Fig. S3d). The above data suggested exosomal miR-31-5p as a biomarker for LUAD.

## Discussion

Hypoxia has been universally considered as a key hallmark of solid tumors [6]. Metastasis, a multifaceted process in LUAD, requires tumor cells to migrate toward regions of sufficient oxygen to strengthen their development, as based on necessary structural changes that need to occur, including EMT [28]. Emerging evidence suggests that hypoxia stress induces the release of exosomes that carry proteins and miRNAs into TME, and thereby influences exosome-mediated intercellular communication [29]. In the present study, we isolated exosomes from the supernatants of hypoxic and normoxic LUAD cells. Combined validation with TEM, NTA and western blotting revealed that hypoxia-induced stress increased the production of exosomes, which is consistent with findings in studies conducted with other malignant tumors [7, 30, 31]. The transfer of exosomes can change the phenotypes of the recipient cells. We observed that exosomes derived from hypoxic cells induced EMT process and enhanced the migration and invasion capabilities of recipient cells. A previous study showed that exosomal miR-23a derived from hypoxic lung cancer cells promoted angiogenesis and vascular permeability [14]. However, the phenotype change that contributed to tumor progression was different from that observed in our study, which suggests that there are other informative biomolecules that greatly influence LUAD.

An increasing body of evidence indicates that miRNAs are major bioactive factors abundantly enriched in exosomes [23, 24]. We compared the miRNA profiles of hypoxic and normoxic exosomes derived from LUAD cells. Initial screening and subsequent verification studies conducted with different types of LUAD cell lines indicated that exosomal miR-31-5p might be a critical factor that influences LUAD development. Exosomal non-coding RNAs can stably exist in body fluids and be protected from degradation by ribonuclease because they are protected by the exosomal membrane [32]. Therefore, we observed that miR-31-5p expression was decreased by treatment with RNase and Triton X-100. Similarity, administration of GW4869 also decreased the levels of miR-31-5p. These data confirmed that miR-31-5p was encapsulated in HExo.

miR-31-5p is ectopically upregulated in various malignant tumors. Previous studies showed that miR-31-5p expression was correlated with lung tumorigenesis and the Warburg effect [33, 34]. However, the effects of miR-31-5p in different space distributions, and especially in exosomes, have not been completely elucidated in LUAD. We discovered that exosomal miR-31-5p could significantly accelerate the metastasis of LUAD cells *in vitro* and *in vivo*, and the effects could be reversed by administration of an miR-31-5p inhibitor. Those findings suggest that miR-31-5p is the bioactive factor in hypoxic exosomes, which induce development of aggressive LUAD phenotypes.

SATB2, an AT-rich binding transcription factor, plays a vital role in tumorigenesis and regulation of gene expression by influencing chromatin structure remodeling [35]. It is well established that SATB2 acts as a negative regulator of EMT in colorectal cancer and non-small-cell lung carcinoma [36]. MiRNAs commonly bind to the 3’UTR of their downstream target mRNA molecules to repress gene expression. We predicted the potential target of exosomal miR-31-5p by conducting a bioinformatics analysis. We subsequently confirmed that SATB2 mRNA was the target of exosomal miR-31-5p by conducting luciferase assays and western blot studies. Notably, knockdown of SATB2 suppressed the migration and invasion of LUAD cells, and that effect was increased by transfection with an miR-31-5p inhibitor. Moreover, studies using the xenograft mouse model further validated our hypothesis. Unfortunately, ROC analysis showed that the synergistic effect of miR-31-5p and SATB2 in LUAD clinics is limited, which indicated that there may exist other axis in controlling LUAD metastasis.

MEK/ERK signaling regulates SATB2-reversed osteogenic differentiation in bone mesenchymal stem cells [37]. To explore the underlying mechanism by which exosomal miR-31-5p mediates tumor progression, we focused on factors which activate MEK/ERK pathway. Surprisingly, our findings showed that upregulation of exosomal miR-31-5p decreased the levels of SATB2 expression and remarkably accelerated EMT process and phosphorylation of MEK and ERK, which aberrantly activates MEK/ERK pathway by addition of miR-31-5p in HExo. Therefore, we believed that hypoxia leads to abundance of miR-31-5p in HExo, which activates MEK/ERK signaling in normoxic cells to promote malignant transformation.

Exosomes released by cancer cells can enter the circulatory system. Recently, informative noncoding RNAs encased in plasma exosomes were isolated and detected [38]. Exosomal miRNAs are strongly correlated with clinicopathological features [27]. Here, our results revealed that the levels of exosomal miR-31-5p were much higher in metastatic patients than in non-metastatic patients, which further validates the metastatic effect of exosomal miR-31-5p on LUAD cells. Moreover, we found that an upregulation of exosomal miR-31-5p was significantly associated with N and TNM stages in LUAD patients, and could be used to discriminate between LUAD patients and healthy control subjects. These findings suggest that exosomal miR-31-5p could serve as a diagnostic biomarker for LUAD. Unfortunately, we were unable to evaluate the prognostic value of exosomal miR-31-5p due to a lack of sufficient follow-up time.

## Conclusions

We provide evidence that miR-31-5p is abundantly enriched in hypoxic LUAD cell-derivedexosomes. Exosomal miR-31-5p plays a critical role in cancer progression by decreasing SATB2 expression and increasing the activity of MEK/ERK pathway. Exosomal miR-31-5p could possibly serve as a circulating diagnostic biomarker for LUAD.

## Supplementary information

**Additional file 1: Fig. S1.** miRNA sequencing of lung hypoxic A549 cells-derived exosomes and normoxic cells-derived exosomes.

**Additional file 2: Fig. S2.** Prediction of miR-31-5p potential target.

**Additional file 3: Fig. S3.** Characterization of plasma-derived exosome from lung adenocarcinoma patients.

Additional file 4.

## Data Availability

The datasets used and/or analyzed during the current study are available from the corresponding author on reasonable request.

## Abbreviations

NSCLC: Non-small cell lung cancer
LUAD: Lung adenocarcinoma
TME: Tumor microenvironment
EMT: Epithelial-mesenchymal transition
HExo: Hypoxic LUAD cell-derived exosomes
NExo: Normoxic LUAD cell-derived exosomes
SATB2: Special AT-Rich Sequence-Binding Protein 2
FBS: Fetal bovine serum
SEC: Size-exclusion chromatography
TEM: Transmission electron microscope
NTA: Nanoparticle tracking analysis
HE: Hematoxylin and eosin
IHC: Immunohistochemistry
WT: Wild type
Mut: Mutant type
ROC: Receiver Operating Characteristic
