# Supplementary figures and images for: Hypoxic tumor-derived exosomal miR-31-5p promotes lung adenocarcinoma metastasis by negatively regulating SATB2-reversed EMT and activating MEK/ERK signaling
Source: J Exp Clin Cancer Res. 2021 Jun 1;40:179. doi: 10.1186/s13046-021-01979-7 (PMC8167983; doi:10.1186/s13046-021-01979-7)

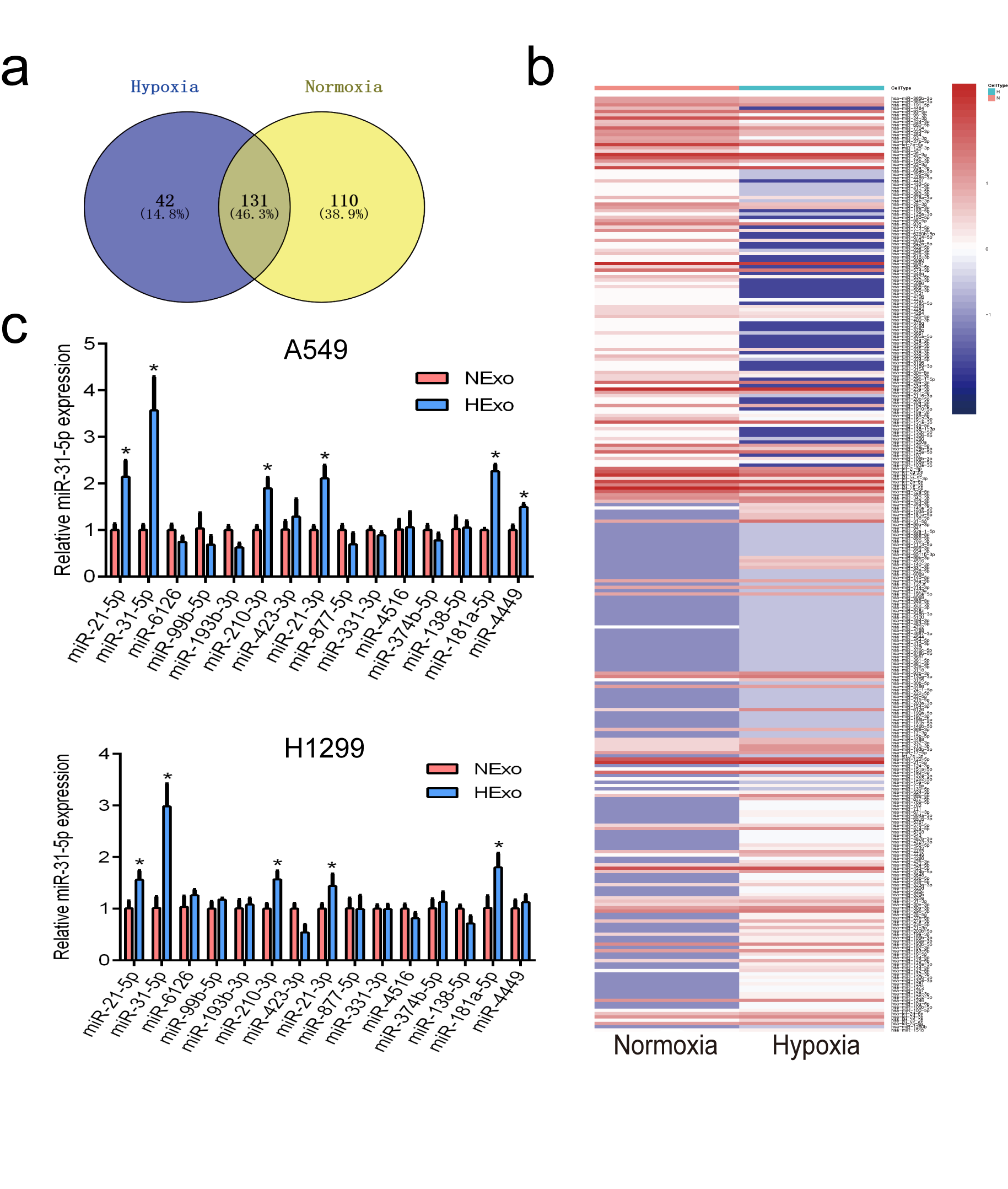

Supplement: Supplementary file 1 — Additional file 1: Fig. S1. miRNA sequencing of lung hypoxic A549 cells-derived exosomes and normoxic cells-derived exosomes. [file 13046_2021_1979_MOESM1_ESM.tif]

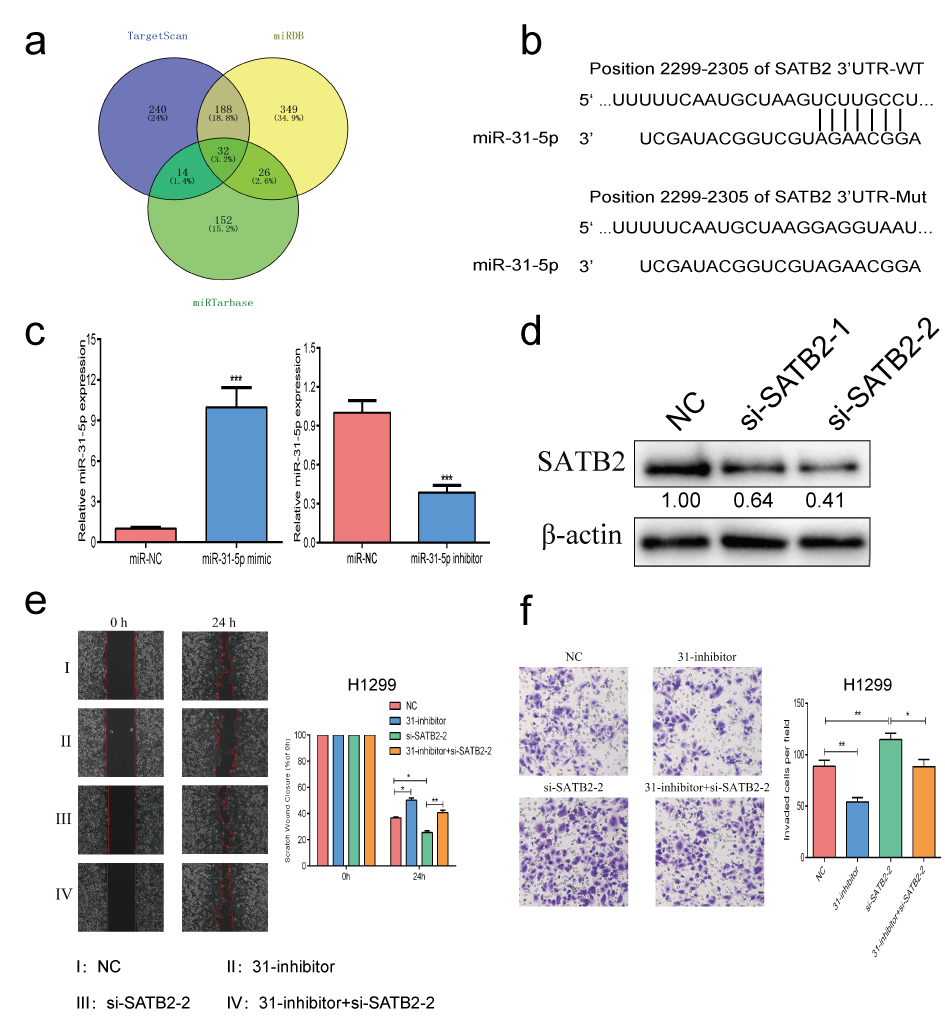

Supplement: Supplementary file 2 — Additional file 2: Fig. S2. Prediction of miR-31-5p potential target. [file 13046_2021_1979_MOESM2_ESM.tif]

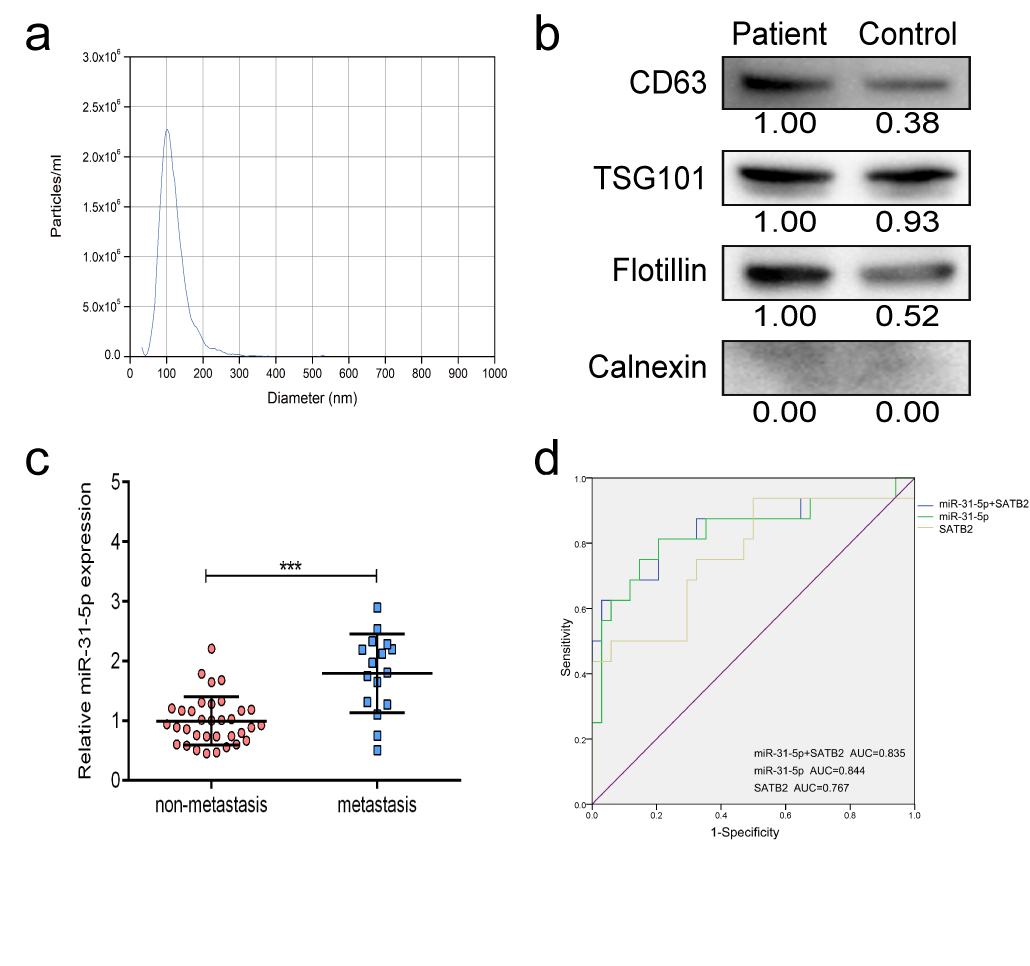

Supplement: Supplementary file 3 — Additional file 3: Fig. S3. Characterization of plasma-derived exosome from lung adenocarcinoma patients. [file 13046_2021_1979_MOESM3_ESM.tif]
